# Conducting a Time Trade-Off Study Alongside a Clinical Trial: A Case Study and Recommendations

**DOI:** 10.1007/s41669-018-0084-1

**Published:** 2018-06-15

**Authors:** Jing Shen, Sarah Hill, David Mott, Matthew Breckons, Luke Vale, Rob Pickard

**Affiliations:** 1 Health Economics Group, Institute of Health and Society, Newcastle University, Newcastle, UK; 2 Office of Health Economics, London, UK; 3 Institute of Cellular Medicine, Newcastle University, Newcastle, UK

## Abstract

Time trade-off (TTO) is an established method in health economics to elicit and value individuals’ preferences for different health states. These preferences are expressed in the form of health-state utilities that are typically used to measure health-related quality of life and calculate quality-adjusted life-years in an economic evaluation. The TTO approach to directly elicit health-state utilities is particularly valuable when generic instruments (e.g. EQ-5D) may not fully capture changes in utility in a clinical trial. However, there is limited guidance on how a TTO study should be conducted alongside a clinical trial despite it being a valuable tool. We present an account of the design and development of a TTO study within a clinical trial as a case study. We describe the development of materials needed for the TTO interviews, the piloting of the TTO materials and interview process, and recommendations for future TTO studies. This paper provides a practical guide and reference for future applications of the TTO method alongside a clinical trial.

## Key Points for Decision Makers

**Table:** 

A time trade-off study is a feasible method to elicit patient preferences and value short-term changes in quality of life alongside a clinical trial.
Extensive piloting and revisions are required when developing the time trade-off materials to ensure they are fit for purpose. The involvement of clinicians and patients during this process is essential.
The complex nature of time trade-off interviews is likely to require face-to-face interviews, and together with an extensive development process, it is important that sufficient time and funding is allocated for the process.

## Introduction

Time trade-off (TTO) is an established method to elicit and value individuals’ preferences for different health states through asking participants to hypothetically trade between quality of life (QOL) and quantity of life [1]. These preferences are measured in the form of utilities that can be used to calculate quality-adjusted life-years (QALYs). QALYs are recommended by the National Institute of Health and Care Excellence (NICE) as the preferred measure of benefit when conducting economic evaluations of healthcare interventions for NHS England and Wales [2]. In an economic evaluation, QALYs are often calculated using generic preference-based QOL measures such as the EQ-5D. The responses to those questionnaires can be translated into QALYs using a pre-determined utility tariff [3–5]. TTO is a key component of health-state valuation protocols in developing utility tariffs for EQ-5D instruments [6, 7]. The EQ-5D is widely used in evaluations of healthcare and public health interventions with advantages of simplicity and brevity compared with other instruments [8]. However, there are instances where the EQ-5D may not capture all relevant gains and losses in health status; for example, within a clinical trial where the fixed frequency of data collection may not capture utility changes resulting from unexpected or short-term acute clinical events. Using a concurrent TTO exercise to capture the (dis-)utility of those clinical events provides a viable alternative. A recent review of the literature found a wide range of procedural considerations when performing TTO tasks [9]; this paper aims to provide a practical guide on the design, development and conduct of a TTO using a worked case study of a TTO alongside a clinical trial [10].

## Time Trade-Off (TTO) Case Study Setting and Methods

The TTO exercise was conducted among a sample of consented eligible trial participants to enhance the economic evaluation within the OPEN (open urethroplasty versus endoscopic urethrotomy—clarifying the management of men with recurrent urethral stricture) study, a UK multicentre randomised trial to determine the clinical and cost effectiveness of open urethroplasty compared with endoscopic urethrotomy in men with recurrent bulbar urethral stricture [10]. The TTO exercise was conducted alongside the trial because the EQ-5D administered at fixed time intervals may not capture short-term but potentially significant decrements in patients’ QOL post-intervention, and they may experience multiple interventions given the likelihood of recurrence of the condition. Those decrements may have significant impact on patients’ QOL that should not be overlooked when conducting cost-utility analysis. The short-term utilities elicited by the TTO will inform the calculation of utilities provided by the EQ-5D.

TTO is typically used to measure the utility of chronic health states where participants remain in the impaired health state for several years. However, in this trial the impaired health states lasted for days or weeks before a return to baseline health. The suitability of the conventional TTO methodology in valuing short-term health states that typically last less than a year has been questioned [11, 12]. Thus, a variant of TTO—chained TTO—has been suggested using an anchor state as a bridge between the temporary states and death [13]. The chained TTO task has two stages: respondents are asked to compare the health-state profiles with the anchor state followed by returning to perfect health (instead of death in a conventional TTO) in stage one, and then compare the anchor state with perfect health state in the conventional TTO task in stage two. In this study, both the conventional and chained TTOs were conducted, and participants were randomly allocated to one of the methods. See Appendix 1 for technical details on the TTO analysis methods.

## Development of TTO Materials

A face-to-face interview format was used because of its anticipated high response rate and the chance to better understand participants’ thought processes. Additionally, because of the complex nature of the chained TTO, it was deemed necessary to administer the study in person rather than use self-completion methods. The materials required to conduct a face-to-face TTO exercise include a TTO board, a number of cards describing health states to be valued and an (optional) interviewer script detailing the process of the TTO exercise. A ‘props method’ (referring to the use of physical interview prompts) [14] was followed to design the materials.

### Decision Board

The decision board was made of an A3 foam board (chosen to withstand extensive use) representing two scenarios: Life A and Life B. The timeline for Life A was fixed while the timeline representing Life B had a movable marker to represent changing time lengths. The timelines enabled participants to visualise the lengths of time spent in each state. The health-state profile cards and the anchor health state were then placed next to Life A and Life B as appropriate.^1^

### Health-State Profiles

The health-state profiles were developed to describe adverse effects of the trial interventions. These ranged from common and mild effects to rare but severe effects. Clinicians and a patient representative were involved throughout the development process to ensure health-state profiles were both medically accurate and understandable. Profiles representing two levels (mild and severe) of adverse effects for each of the two trial interventions were developed, resulting in a total of four health profiles to be valued. The burden on participants to value four health profiles was considered low compared with other studies [12, 15, 16]. These profiles were printed on laminated A6-sized coloured cards.

### Anchor State

The anchor state profile required for the chained TTO describes a hypothetical situation that is intuitively worse than the most severe health profile being valued yet preferable to death, in order to enable trade-off in the first stage of the exercise. The suitability of the anchor state required substantial testing based on reported problems in previous studies [17, 18]. The anchor state was printed on a laminated A6-sized card.

### Time Horizon

One of the challenges in the design of a TTO exercise is to decide on an appropriate time horizon. The condition-specific adverse effects in the OPEN study ranged from a few days to several months. A duration of 28 days was initially chosen based on the longest duration of short-term adverse effects (those lasting < 1 year). Within each health profile, specific lengths of time were stipulated for adverse effects that did not last for the entire 28 days. The intention was to give a realistic representation of the impaired health states immediately following the trial interventions.

### Script and Training Video

We produced an interview script with instructions for each version of the TTO exercise. As study participants were recruited across the UK, it was decided to enlist research nurses (RNs) at participating trial sites to conduct a proportion of the TTO interviews. A training video was produced to provide RNs with an additional resource to supplement face-to-face training by the study team.

The interview script provided suggested wording for interviewers to use during the TTO exercise. Creating an interview script ensures all interviews are conducted consistently. We observed that after several interviews it was not necessary to read verbatim from the script, but it remained a useful point of reference and ensured fidelity of the interview process. The interview scripts and instructions are included in Appendix 3.

The training video was structured as a ‘mock’ interview, and covered common difficulties encountered in a TTO exercise (Appendix 4).

## Piloting of TTO Materials and Process

**Fig. 1:**
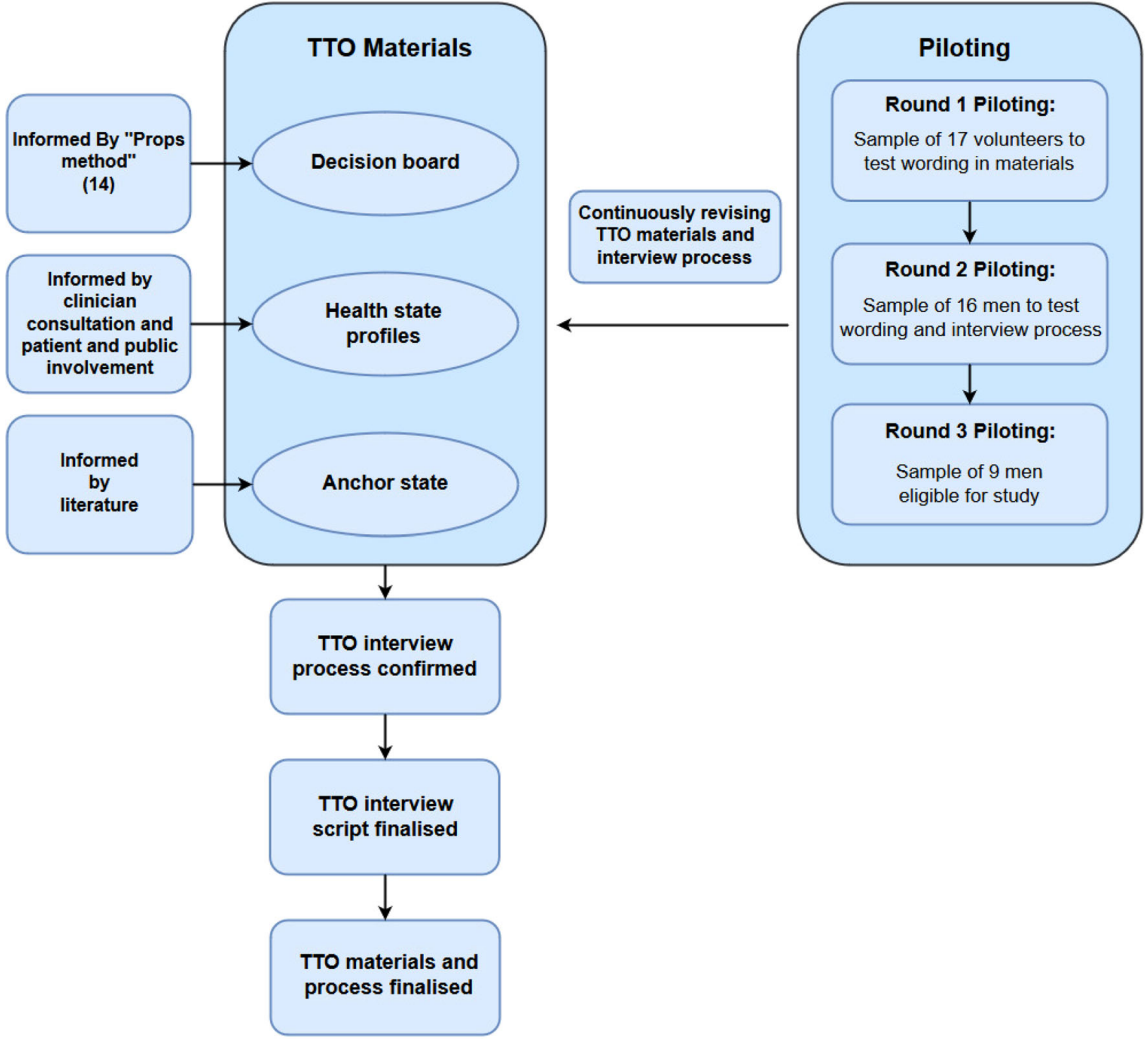
Time trade-off (TTO) exercise development

We conducted three rounds of piloting of materials and processes; following each round, the project team discussed the issues noted, refining materials and processes where appropriate. This process is described in Fig. 1.

### Round 1

Piloting of the initial versions of the TTO materials was conducted with 17 male and female volunteers (researchers at Newcastle University); the aim here was to ensure plain language was used. During this round, it quickly became clear that participants often entered the TTO exercise without fully understanding the task ahead. A decision was made to add a practice task to ‘warm-up’ participants, as suggested in previous studies [6, 19]. Generic EQ-5D-3L profiles were used in the practice task. For the conventional TTO method, one practice health state was evaluated before the start of the main TTO exercise. For the chained TTO method, one practice health state was evaluated before each stage of the chained process. Alterations to the materials were made following this first round of piloting and tested in subsequent piloting.

### Round 2

Piloting was conducted with 15 male staff members from a participating study site and a patient representative. This round was limited to men because we wanted to test the TTO materials with the gender of interest for this study. These pilots were used to assess whether the changes made in response to the first round of piloting were sufficient or if further amendments were necessary.

### Round 3

Piloting was conducted with nine men eligible for the OPEN trial. Review of the resulting data assessed internal consistency of the health-state valuations and found evidence that participants might still not fully understand the TTO exercise. A major change to the TTO exercise was made to extend the practice period by adding an extensive practice exercise (described in the pre-interview practice section below).

Piloting was crucial to the development of the TTO exercise, reflected by the resulting feedback and revisions, and also served as a training exercise for researchers. Sufficient time should be allowed for piloting before embarking on the study data collection. Efforts should be made to ensure piloting includes individuals with similar characteristics to the target study participants, so that potential issues relating to the characteristics of study participants can be identified. Following piloting, the following revisions were made:

### Health-State Profiles

Piloting showed that the burden of valuing four profiles was low. With further clinical input it was therefore decided to further divide adverse effects into mild, moderate and severe, „ in total (Appendix 2). More profiles allowed a richer data set to estimate health-state utilities and the nuances of each health state were better expressed.

### Anchor State

As noted above, a chained TTO requires an anchor state. The first version of the anchor state was titled ‘chronic pain’ and described a health state in which the individual experienced chronic, debilitating pain with no relief. Piloting identified two problems with this profile: firstly, ‘chronic’ implied long term, which was at odds with the short duration of the profile; secondly, the health state described was perceived as worse than being dead by a large proportion of piloting participants. The title of the anchor state was accordingly changed to ‘severe pain’ to imply less permanency and the severity was reduced: maintaining the impairment of usual functioning (i.e. working, leisure activities), but allowing basic self-caring activities. This was considered in most subsequent piloting as a state between the most severe of the health states being valued and death. This was because participants felt that maintaining basic functioning without complete dependency on others was important for a health state to be considered better than death. The final version of the anchor state is in Appendix 2.

### Time Horizon

Piloting identified problems in the original time horizon format where the entire health state lasted 28 days, but within this some components had their own specific timelines, such as an overnight hospital stay. These different timeframes appeared confusing for participants and re-evaluation of the profiles led us to implement a reduced time horizon of 14 days. Adverse effects that lasted fewer days were specified as such within the profiles and were defined in relatively non-specific terms within the descriptions as ‘a few days’ or ‘overnight’; the intention was to minimise cognitive burden on participants by providing only one numerical value (14 days) to concentrate on when considering each health state. Piloting confirmed that participants preferred this format. The more complex a health-state profile is, the more difficult it is for participants to visualise and evaluate. Piloting emphasised the need to ensure an appropriate balance between realistic and clinically accurate health-state profiles, and simple and clear scenarios.

### Pre-interview Practice

Piloting demonstrated that an extended practice period was needed to improve participants’ understanding of the TTO exercise. In the extended practice, participants were asked to evaluate three health-state profiles (based on EQ-5D profiles) as examples after ranking them from the best to worst. Participants’ evaluations on the practice health profiles were then immediately compared with their rankings using a ‘Practice Sheet’,^2^ and they were also asked to verbalise their decision-making process. The researcher then talked through the practice results with the participants using the ‘Practice Sheet’ as a discussion tool, giving them a chance to reflect on their decisions and ask questions.

## Steps of TTO Interviews

**Fig. 2:**
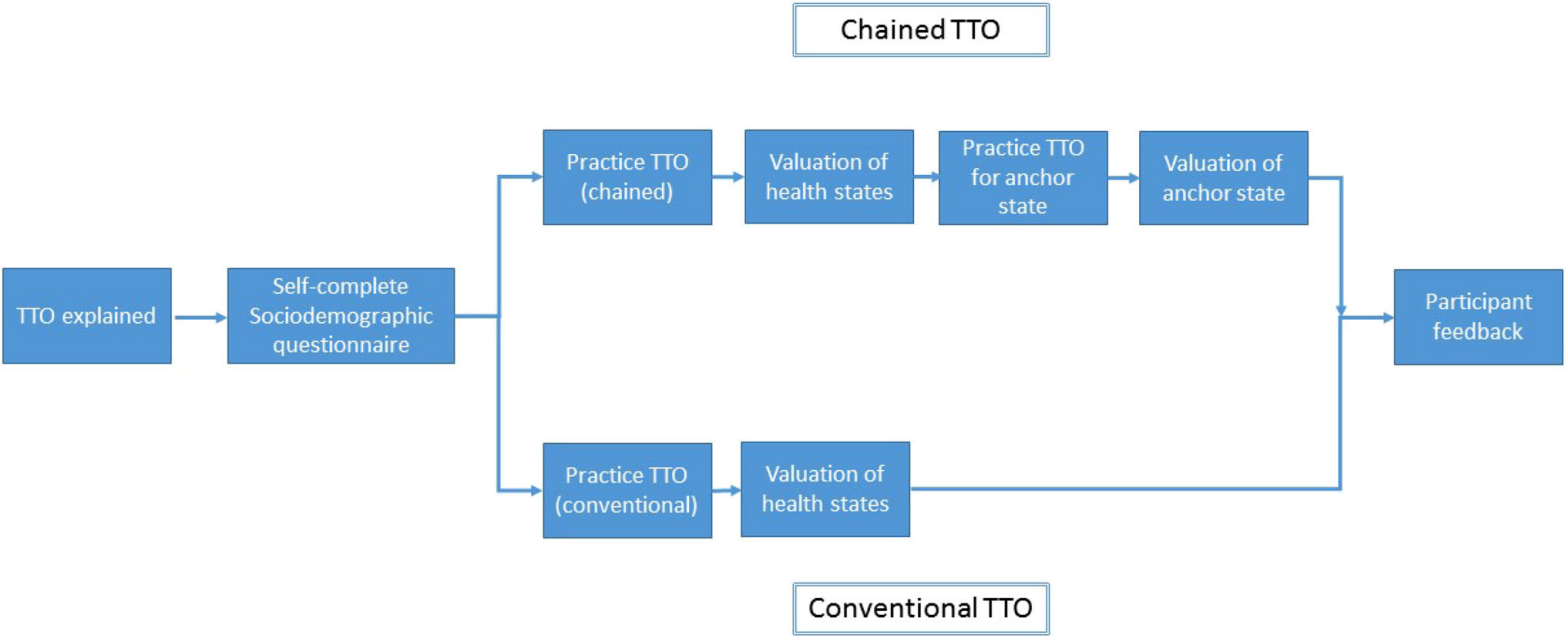
Time trade-off (TTO) exercise procedure

Following piloting, the TTO interview procedure was agreed (Fig. 2). Ethical approval was obtained as part of the OPEN study. Upon consenting to the TTO interview, participants were informed about the aim of the study and the interview process, following which they were asked to complete a short questionnaire regarding their sociodemographic details before the TTO exercise began. At the end of the TTO exercise, participants were asked to rate the difficulty of the task on a 1–5 scale (1 being ‘no difficulty at all’ and 5 being ‘very difficult’) and provide additional feedback if they wished. Following completion of the interview, interviewers noted their own reflections on the process.

## The TTO Exercise

**Fig. 3:**
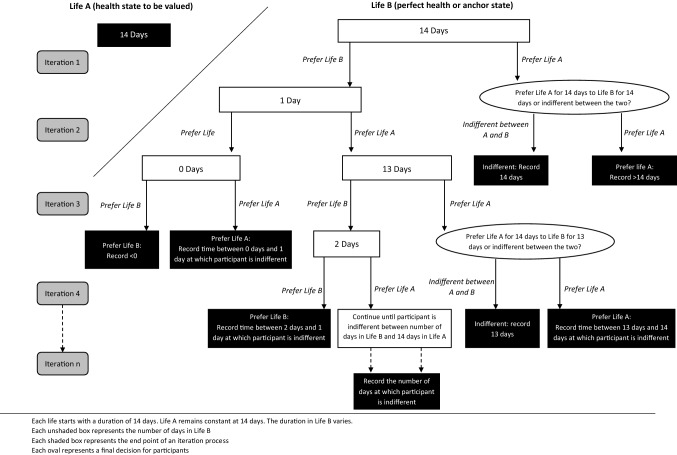
Iteration process of varying time in Life B in the time trade-off exercise

An illustration of the interview process is provided in Fig. 3. In order to obtain a utility value for each health state, the TTO exercise aims to elicit a point at which a participant is indifferent between the health state being valued (Life A) and an alternative state (Life B). Initially the participant is asked to state a preference for Life A or Life B when the duration of each is 14 days (Fig. 3; Iteration 1). This is followed by an iterative process of varying the time a participant ‘spends’ in Life B from 14 days to 1 day to 13 days to 2 days, etc. (Figure 3; Iterations 2–4) in order to ascertain the duration of time in the alternative state which is equivalent to 14 days in Life A; this number of days is recorded by the researcher. This process will continue until the participant is indifferent between Life A and Life B. Figure 3 provides a visual representation of the interview process described in the full interview script, which is included in Appendix 3.

## Results of TTO Interviews

Overall, 40 participants were recruited to the study; 20 participants completed each TTO variant. Two participants did not complete the TTO tasks, and were excluded from the analysis. The median difficulty rating was 2 (range 1–5), suggesting the TTO exercise was viewed as reasonably easy. Participants described their decision-making process, which drew on personal life experiences, family situations and experiences of the interventions being evaluated. Adaptation was observed as some participants stated that after having experienced urinary symptoms and the use of a urinary catheter, they no longer felt they would be as negatively impacted by a recurrence. Interviewer notes generally covered how well the researcher felt the participant had understood the task. In all but a minority of cases, the researcher perceived that the participant had understood the task. Interviewers noted where participants had given apparently illogical answers and any possible reasons for this. Those qualitative elements of the TTO exercise were useful for interpreting the health-state valuations results.

## Discussion and Reflections

This paper provides a practical guide on the development, design and conduct of a TTO study (Fig. 1). We believe it makes a valuable contribution to the literature given the paucity of practical descriptions of conducting TTO studies. The illustrative case study was part of a clinical trial comparing two surgical interventions. Both of the interventions led to short-term disutility that may not be captured by generic preference-based QOL measures. This setting represents an example of the value of a TTO exercise in such circumstances. This paper provides a detailed account of the TTO exercise, including how study materials were developed, how iterative piloting informed changes, and gives our reflections and recommendations for researchers planning to use TTO.

A number of recommendations for best practice in designing and conducting a TTO exercise are made based on study reflections. In the design stage of a TTO exercise, if health states are based on a clinical scenario, the involvement of clinicians in developing the profiles is essential. Clinicians are experienced at translating textbook clinical effects into lay language; therefore, their involvement ensures health-state profiles are accurate and realistic, yet simple and clear to participants. For the purpose of the case study, the descriptions of health-state profiles were based only on adverse effects of the interventions, however, it should be noted that in the design of health-state profiles, both negative and positive aspects of health may be included dependent on the study question. Additionally, patient and public involvement (PPI) throughout the design and piloting phases was essential to ensure the final product was fit for purpose. In the development of a TTO exercise, multiple rounds of extensive piloting are crucial to ensure the study materials and processes are fit for purpose. It is also essential to pilot using a sample with similar characteristics to the target population. A further key message from our work is the need to conduct an extensive practice task before the main TTO exercise. This was particularly useful in ensuring participants fully understand the logic behind the TTO exercise before engaging in the evaluation of study health-state profiles. Another point to note is that TTO interviews, if conducted face-to-face and at geographically distant locations, are resource intensive; sufficient time and funding should be allocated for both the development and conduct of the TTO study.

## Conclusion

In summary, this paper provides practical guidance and recommendations for conducting a TTO study alongside a clinical trial. Given the value and potential broad usage of TTO in measuring QOL, we believe this paper will be a useful resource for researchers who wish to embark on designing a TTO exercise in their own studies.

## Appendix 1: Technical Details of the Time Trade-Off (TTO) Methods

### The Conventional Method

In a conventional TTO exercise, respondents are offered a choice between two alternative health states at a time—one is the less desirable health state (*h*_i_) and the other is the perfect health state. The time spent in health state *h*_i_ is fixed at *t* whereas the length of time (between 0 and *t*) spent in the perfect health state varies, and both are followed by death. Respondents are asked to imagine themselves in both of the scenarios and find a point (time *x*) that they are indifferent to the lengths of time spent in health state *h*_i_ (*t*) and perfect health (*x*). The same process is done for each health state to be valued in the study. The utility value of each health state (*h*_i_) is then calculated as: $h_{\text{i}} = x/t.$

### The Chained Method

The chained TTO comprises two stages: in the first stage, participants are asked to compare the temporary health states with the anchor state. The anchor state must be worse than the temporary health state, but better than death. Time spent in the temporary state is fixed at *t* whereas the time period of the anchor state is varied, both followed by a return to perfect health. Participants are asked to imagine themselves in both of the scenarios and find a time point (*X*_1_) between 0 and *t* for the anchor state where they are indifferent to being in either of the two scenarios. In the second stage, the anchor state will be valued in a conventional TTO exercise where participants are asked to compare the anchor state and a perfect health state. The time period in the perfect health state is varied between 0 and *t* whereas the anchor state is fixed at t, both followed by death. Participants are asked to imagine themselves in both of the scenarios and find a time point (*X*_2_) between 0 and *t* for the perfect health state where they are indifferent in being in either of the two scenarios. The utilities of the temporary health states being valued will be calculated based on *X*_1_ and *X*_2_.

The utility value for each of the health profiles developed are calculated as follows, where h_i_ is the utility value for the temporary health states and h_j_ is the utility value for the anchor state.

$$ h_{\text{i}} = 1 - \left( {1 - h_{\text{j}} } \right)\frac{{x_{1} }}{t} $$

$$ h_{\text{j}} = \frac{{x_{2} }}{t}. $$

Combining the above, the formula for calculating the utility value for each of the health profiles is:

$$ h_{\text{i}} = 1 - \left( {1 - \frac{{X_{2} }}{t}} \right)\frac{{X_{1} }}{t}. $$

## Appendix 2: Health State Profiles Used in the Study

### Control Intervention: Urethrotomy Health State Profiles

**Table:** Urethrotomy: Mild

Discomfort in the penis and bladder from using a catheter for a few days
Brief discomfort on passing urine after the catheter is removed
A few drops of blood after you have finished passing urine
Mild urinary tract infection giving you mild fever-like symptoms

**Table:** Urethrotomy: Moderate

Discomfort in the penis and bladder from using a catheter for a few days
Discomfort on passing urine after the catheter is removed
Moderate urethral bleeding which requires you to keep the catheter in longer or have a telescopic examination under anaesthetic
Serious urinary tract infection which makes you feel ill and requires you to stay in hospital overnight for antibiotics from an IV drip

**Table:** Urethrotomy: Severe

Discomfort in the penis and bladder from using a catheter
Severe urethral bleeding which requires you to have a telescopic examination under anaesthetic
Serious urinary tract infection which makes you feel ill and requires you to stay in hospital overnight for antibiotics from an IV drip
Severe pain in the penis and bladder area requiring you to take regular painkillers
Difficulty getting and maintaining a penile erection for sex

### Experimental Intervention: Urethroplasty Health-State Profiles

**Table:** Urethroplasty: Mild

Discomfort in the penis and bladder from using a catheter
Mild mouth pain or discomfort when you eat or drink
Mild urinary tract infection giving you mild fever-like symptoms
Mild swelling and wound pain in the area between the testes and back passage

**Table:** Urethroplasty: Moderate

Discomfort in the penis and bladder from using a catheter
Moderate and constant mouth pain and scarring in the mouth needing regular painkillers
Serious urinary tract and wound infection which makes you feel ill and requires you to stay in hospital overnight for antibiotics from an IV drip
Moderate wound pain in the area between the testes and back passage needing regular painkillers

**Table:** Urethroplasty: Severe

Discomfort in the penis and bladder from using a catheter
Severe and constant mouth pain and scarring in the mouth needing regular painkillers
Serious urinary tract and wound infection which makes you feel ill and requires you to stay in hospital overnight for antibiotics from an IV drip
Severe wound pain in the area between the testes and back passage needing regular painkillers
Leakage of urine from the area between the testes and back passage requiring you to wear incontinence pads
Difficulty getting and maintaining a penile erection for sex

**Table:** Anchor State

*Severe pain state*
You have recently been injured and as a result of the injury:
You are able to do basic tasks (e.g. washing, feeding and communicating) but you have problems walking about
You have extreme pain and discomfort. No medication can completely alleviate the pain
You cannot take part in usual activities (e.g. work, social activities and exercise)

## Appendix 3: TTO Interview Scripts and Instructions

### Conventional TTO interview script (underline) and instructions

#### Introduction

Interviewer introduce oneself and thank the participant for their time. Indicate that it is expected to spend up to 1 hour on this interview. Ask the participant if they have any questions before the interview begins. Take written consent from the participant.

Ask the participant to fill in the consent form and the ‘TTO Study—Data Collection Form’. Let the participant know that the data collection form is just to provide details which will help with the analysis later on. All details will be kept anonymous and confidential.

Reiterate that the interview is voluntary and can finish at any time that they wish.

#### Format and Purpose of the interview

The interview we will be doing today is a little different to any interview you may have done before, but don’t worry we will explain everything fully as we go along and run through a practice at the beginning to make sure you are comfortable with the task. What we will be doing today is an activity called a time trade off exercise which is a tool used by health economists to put a number on how you value a particular state of health. We are going to use this board [point at the TTO board] throughout the interview and each of these cards is going to describe and represent a particular state of health. I will call these cards health states. We are going to ask you to imagine yourself in these health states and think about how you may feel in a situation where your heath is as it is described on the card. We are now going to do a practice run to help you understand how the interview works. There are no right or wrong answers in this practice session.

#### Practice Exercise

I am first going to show you three health states. These are just general health states and do not bear any reflection on your actual health at the moment. I would like you to imagine how you might feel and be affected if your health was as described on each card.

Ask the participant to read the ‘practice profiles’ out loud and ask them to rank the health states from the best to the worst state. Remind the participant that this is not ‘most like their health’ but is simply the best to worst at face value of each card. Write down the order chosen by the participant (they are labelled on the back of the card A–C).

Describe the task: On the board there are two lives A and B. ‘Life A’ is going to last for 14 days—this won’t change during the interview. The other health state or ‘Life B’ will vary in how long it lasts—up to a maximum of 14 days.

Place one of the practice cards opposite the marker for ‘Life A’. In Life A you should imagine that your health is as described in this health state. You will be in that health state for 14 days then at the end of the 14 days you will die a quick and painless death.

Place the ‘perfect health’ card opposite the marker for ‘Life B’. In Life B you will have perfect health. The length of time you spend in this health state will vary throughout the interview but same as in Life A, at the end of time in this health state, you will die a quick and painless death.

Ask the participant to read these health states again and to try to imagine themselves in these health states.

Move both markers to 14 days and ask the participant the following question: Would you prefer ‘Life B’, perfect health, for 14 days followed by a quick and painless death or would you prefer ‘Life A’ for 14 days followed by a quick and painless death?

Presumably, the participant will make the logical decision to choose Life B. Therefore, move the marker next to ‘Life B’ to 1 day and ask the participant the following question: Would you prefer 1 day in ‘Life B’ followed by a quick and painless death or would you prefer ‘Life A’ for 14 days followed by a quick and painless death? Or would you not be able to choose between the two?

Continue asking this question for the scenario where ‘Life B’ lasts for 13 days, 2 days, 12 days, 3 days, 11 days, 4 days, 10 days, 5 days, etc. until the participant cannot decide between the two health states. You may get to a point where at x days in Life B (for example, 4 days) they prefer Life B but an increase in 1 day (to 5 days) switches their preference so that they now prefer Life A. At this point you will expect the point where they cannot choose to lie somewhere between 4 and 5 days (the points at which their preferences changed). Ask the participant if they think the point at which they cannot choose between Life A or Life B is one of those numbers of days (4 or 5 in this example) or somewhere in between. Try not to suggest numbers to them but let them know they can half or part of a day should they wish.

Note down the number of days the participant is willing to give up in ‘Life B’, perfect health, to avoid 14 days in Life A on the practice sheet and on the practice sheet. (i.e. 14 minus the number of days at which the participant could not decide between the two health states).

Repeat this task for the remaining two practice states.

The practice sheet will allow you to assess if the participant understands the task and is providing logical responses. First of all, place the health state cards on the practice sheet in the order the participant ranked them. Then underneath each card write the number of days they were willing to be in Life B that corresponds with that card. These are the numbers you will have written down on the data collection sheet. You are looking for logical responses i.e. the number of days in Life B should increase (or at least not decrease) from the best to worst health states. If the responses don’t seem logical, remember there are no right or wrong answers but ask the participant if they thinks their responses make sense looking at them on the practice sheet. Ask them if they would change any of their responses while talking them through what their responses mean. Spend as much time as necessary until the participant understands the TTO process.

#### Symptom Specific Profiles

Following the practice session, we now move onto the main part of the TTO task, evaluating health states associated with the adverse-effect of the interventions in the trial. Explain to the participant that they do not have to have had any of these symptoms described in the health state profiles, they just need to imagine himself being in these health states.

Ask the participant to read the health state profiles and rank the health states from the best to the worst. Write down the order chosen by the participant. Let them know they can take their time at this point as it is essential they read through all the cards thoroughly.

Explain that the task will work in exactly the same way as the practice tasks: On the board, one health state or ‘Life A’ is going to last for 14 days—this will not change. The other health state or ‘Life B’ will vary in how long it lasts—up to a maximum of 14 days.

Shuffle the cards so that they are not in the order the participant ranked them. Let the participant know you are doing this and that the cards will be presented in a random order. Place the first profile card opposite the marker for ‘Life A’.

Place the ‘perfect health’ card opposite the marker for ‘Life B’.

Ask the participant to read these health states again out loud and to try to imagine themselves in these health states.

Move both markers to 14 days and ask the participant the following question: Would you prefer ‘Life B’, perfect health, for 14 days followed by a quick and painless death or would you prefer ‘Life A’ for 14 days followed by a quick and painless death?

Presumably, the participant will make the logical decision to choose Life B. Therefore, move the marker next to ‘Life B’ to 1 day and ask the participant the following question: Would you prefer 1 day in ‘Life B’ followed by a quick and painless death or would you prefer ‘Life A’ for 14 days followed by a quick and painless death? Or would you not be able to choose between the two?

Continue asking this question for the scenario where ‘Life B’ lasts for 13 days, 2 days, 12 days, 3 days, 11 days, 4 days, 10 days, 5 days, etc. until the participant cannot decide between the two health states. Follow the same procedure as in the practice for identifying the number of days in perfect health that are equivalent to 14 days in Life A.

Note down the number of days at which the participant is indifferent between the two health states on the data collection sheet.

Repeat this task for the remaining five profiles.

#### End the interview

At the end of the TTO process, ask the participant about how difficult they rate the interview task and any comments they have on the interview. Conclude the interview and thank the participant.

### Chained TTO interview script (underline) and instructions

#### Introduction

Interviewer introduce oneself and thank the participant for their time. Indicate that it is expected to spend up to 1 hour on this interview. Ask the participant if they have any questions before the interview begins. Take written consent from the participant.

Ask the participant to fill in the consent form and the ‘TTO Study—Data Collection Form’. Let the participant know that the data collection form is just to provide details which will help with the analysis later on. All details will be kept anonymous and confidential.

Reiterate that the interview is voluntary and can finish at any time that they wish.

#### Format and Purpose of the interview

The interview we will be doing today is a little different to any interview you may have done before, but don’t worry we will explain everything fully as we go along and run through a practice at the beginning to make sure you are comfortable with the task. What we will be doing today is an activity called a time trade off exercise which is a tool used by health economists to put a number on how you value a particular state of health. We are going to use this board [point at the TTO board] throughout the interview and each of these cards is going to describe and represent a particular state of health. I will call these cards health states. We are going to ask you to imagine yourself in these health states and think about how you may feel in a situation where your heath is as it is described on the card. We are now going to do a practice run to help you understand how the interview works. There are no right or wrong answers in this practice session.

#### Practice Exercise

I am first going to show you three health states. These are just general health states and do not bear any reflection on your actual health at the moment. I would like you to imagine how you might feel and be affected if your health was as described on each card.

Ask the participant to read the ‘practice profiles’ out loud and ask them to rank the health states from the best to the worst state. Remind participant that this is not ‘most like their health’ but is simply the best to worst at face value of each card. Write down the order chosen by the participant (they are labelled on the back of the card A-C).

Describe the task: On the board there are two lives A and B. ‘Life A’ is going to last for 14 days—this won’t change during the interview. ‘Life B’ will vary in how long it lasts—up to a maximum of 14 days.

Place one of the practice cards opposite the marker for ‘Life A’. In Life A you should imagine that your health is as described in this health state. You will be in that health state for 14 days then at the end of the 14 days you will return to full health.

Place the anchor state card opposite the marker for ‘Life B’. In Life B your health will be as it is described in this anchor state card. The length of time you spend in this health state will vary throughout the interview but as in Life A you will always return to full health at the end of the time.

Ask the participant to read these health states again and to try to imagine themselves in these health states.

Move both markers to 14 days and ask the participant the following question: Would you prefer ‘Life B’, the anchor state, for 14 days followed by a full recovery or would you prefer ‘Life A’ for 14 days followed by a full recovery?

Presumably, the participant will make the logical decision to choose Life A. If they do, move the marker next to ‘Life B’ to 1 day and ask the participant the following question: Would you prefer 1 day in ‘Life B’ followed by a full recovery or would you prefer ‘Life A’ for 14 days followed by a full recovery? Or would you not be able to choose between the two?

Continue asking this question for the scenario where ‘Life B’ lasts for 13 days, 2 days, 12 days, 3 days, 11 days, 4 days, 10 days, 5 days, etc. until the participant cannot decide between the two health states. You may get to a point where at x days in Life B (for example, 4 days) they prefer Life B but an increase in 1 day (to 5 days) switches their preference so that they now prefer Life A. At this point you will expect the point where they cannot choose to lie somewhere between 4 and 5 days (the points at which their preferences changed). Ask the participant if they think the point at which they cannot choose between Life A or Life B is one of those numbers of days (4 or 5 in this example) or somewhere in between. Try not to suggest numbers to them but let them know they can half or part of a day should they wish.

Note down the number of days the participant is willing to be in ‘Life B’, the anchor state, to avoid 14 days in Life A on the practice sheet.

Repeat this task for the remaining two practice states.

The practice sheet will allow you to assess if the participants understand the task and are providing logical responses. First of all place the health state cards on the practice sheet in the order the participant ranked them. Then underneath each card write the number of days they were willing to be in Life B that corresponds with that card. These are the numbers you will have written down on the data collection sheet. You are looking for logical responses i.e. the number of days in Life B should increase (or at least not decrease) from the best to worst health states. If the responses do not seem logical, remember there are no right or wrong answers but ask the participant if they think their responses make sense looking at them on the practice sheet. Ask them if they would change any of their responses while talking them through what their responses mean. Spend as much time as necessary until the participant understands the TTO process.

#### Symptom Specific Profiles

Following the practice session, we now move onto the main part of the TTO task, evaluating health states associated with the adverse-effect of the interventions in the trial. Explain to the participant that they do not have to have had any of these symptoms described in the health state profiles, they just needs to imagine themselves in these health states with these particular symptoms.

Ask the participant to read the health state profiles and rank the health states from the best to the worst. Write down the order chosen by the participant. Let them know they can take their time at this point as it is essential they read through all the cards properly and thoroughly.

Explain that the task will work in exactly the same way as the practice tasks: On the board, one health state or ‘Life A’ is going to last for 14 days—this will not change. The other health state or ‘Life B’ will vary in how long it lasts—up to a maximum of 14 days.

Shuffle the cards so that they are not in the order the participant ranked them. Let the participant know you are doing this and that the cards will be presented in a random order. Place the first profile card opposite the marker for ‘Life A’.

Place the anchor state card opposite the marker for ‘Life B’.

Ask the participant to read these health states again out loud and to try to imagine themselves in these health states.

Move both markers to 14 days and ask the participant the following question: Would you prefer ‘Life B’, the anchor state, for 14 days followed by a full recovery or would you prefer ‘Life A’ for 14 days followed by a full recovery?

Presumably, the participant will make the logical decision to choose Life A. Therefore, move the marker next to ‘Life B’ to 1 day and ask the participant the following question: Would you prefer 1 day in ‘Life B’ followed by a full recovery or would you prefer ‘Life A’ for 14 days followed by a full recovery? Or would you not be able to choose between the two?

Continue asking this question for the scenario where ‘Life B’ lasts for 13 days, 2 days, 12 days, 3 days, 11 days, 4 days, 10 days, 5 days, etc. until the participant cannot decide between the two health states. Follow the same procedure as in the practice for identifying the number of days in the anchor state that are equivalent to 14 days in Life A.

Note down the number of days the participant is willing to be in ‘Life B’, the anchor state, to avoid 14 days in Life A on the data collection sheet.

Repeat this task for the remaining five profiles.

#### Valuing the Anchor State

The last task is to work out the participant’s preference for the anchor state. This requires another practice task as this is slightly different from the previous tasks.

As before, on the board, one health state or ‘Life A’ is going to last for 14 days—this will not change. However, at the end of the 14 days rather than recovering you will die a quick and painless death. The other health state or ‘Life B’ will vary in how long it lasts—up to a maximum of 14 days and again at the end of the time you will die a quick and painless death.

Place one of the practice profile cards opposite the marker for ‘Life A’.

Place the ‘perfect health’ card opposite the marker for ‘Life B’.

Ask the participant to read these health states again and to try to imagine themselves in these health states. Perfect health is anything the participant believes it to be.

Move both markers to 14 days and ask the participant the following question: Would you prefer ‘Life B’, perfect health, for 14 days followed by a quick and painless death or would you prefer ‘Life A’ for 14 days followed by a quick and painless death? This is an unusual question to ask so please let the participant know that it is understandable for it to be difficult to imagine but ask them to try.

Presumably, the participant will make the logical decision to choose Life B. Therefore, move the marker next to ‘Life B’ to 1 day and ask the participant the following question: Would you prefer 1 day in ‘Life B’ followed by a quick and painless death or would you prefer ‘Life A’ for 14 days followed by a quick and painless death? Or would you not be able to choose between the two?

Continue asking this question for the scenarios where ‘Life B’ lasts for 13 days, 2 days, 12 days, 3 days, 11 days, 4 days, 10 days, 5 days, etc. until the participant cannot decide between the two health states.

Note down at what day the participant is indifferent between the two health states on the practice sheet.

This is the end of the practice task, ask the participant if they understand the task, if they have any questions and if they are happy to move on.

#### To Value the Anchor State

Place the anchor state opposite the marker for ‘Life A’.

Place the ‘perfect health’ card opposite the marker for ‘Life B’.

Ask the participant to read these health states again and to try to imagine themselves in these health states.

Move both markers to 14 days and ask the participant the following question: Would you prefer ‘Life B’, perfect health, for 14 days followed by a quick and painless death or would you prefer ‘Life A’ for 14 days followed by a quick and painless death?

Presumably, the participant will make the logical decision to choose Life B. Therefore, move the marker next to ‘Life B’ to 1 day and ask the participant the following question: Would you prefer 1 day in ‘Life B’ followed by a quick and painless death or would you prefer ‘Life A’ for 14 days followed by a quick and painless death? Or would you not be able to choose between the two?

Continue asking this question for the scenarios where ‘Life B’ lasts for 13 days, 2 days, 12 days, 3 days, 11 days, 4 days, 10 days, 5 days, etc. until the participant cannot decide between the two health states.

Note down at what day the participant is indifferent between the two health states on the Collection sheet.

#### End the interview

At the end of the TTO process, ask the participant about how difficult they rate the interview task and any comments they have on the interview. Conclude the interview and thank the participant.

## Appendix 4: Dealing with Common Problems when Conducting a TTO

*Common issue one: Participants benchmark health states being valued against their current health*

There was a tendency for participants to relate the health profiles being valued to their own health and this could result in participants ranking health profiles by how much they resemble their own situation.

*Solution*: When the interviewer observes participants displaying this tendency during the practice section, the interviewer should re-emphasise that this exercise requires the participant to consider each health state at face value hypothetically and not in relation to their own health.

*Common Issue Two:* *Reaching Equivalency*

It has been observed that it can sometimes be difficult for participants to reach a point of indifference. For example, a participant would have a clear preference for 6 days in Life B over 14 days in Life A, however, once the time in Life B is reduced by just one day to 5 days, they would switch to prefer 14 days in Life A over 5 days in Life B.

*Solution*: When participants appear to be unable to reach a point of indifference, the interviewer would first verbalise in lay language the preferences they showed on the decision board, for example, “at this current point [refereeing to the slider on the decision board], that is 6 days spent in Life B, you think that is better than 14 days in Life A?” and once the participant confirms it, the interviewer would then move the slider to a different point and repeat “at this current point [referring to the slider on the decision board], that is now 5 days spent in Life B, you think that is worse than 14 days in Life A?” The verbalisation of their choices would sometimes help the participants to find an equivalent point. If they still unable to find an indifference point, the interviewer would suggest them choosing fractions of days and asked if they felt that there is a value in between these durations (5 or 6 days), for example, 5 ¼, 5 ½ or 5 ¾ days if they wish.

*Common issue three: Valuing the Anchor state*

Due to the severity of the anchor state which is purposely designed to be intuitively worse than all the states being valued, participants may be unwilling to trade as they consider any time in the perfect health state is better than 14 days in the anchor state, which sometimes lead to them prefer 0 day in perfect health to 14 days in the anchor state. As we purposely design the anchor state to be not worse than being dead, this creates a difficult situation.

*Solution*: While we would tell participants that the choice of spending 0 days in the perfect health state is perfectly acceptable, we would also remind them that 0 days in perfect health equates to ‘instant death’ and ask the participant whether they consider ‘instant death’ equivalent to 14 days in the anchor state. This may help the participants re-evaluate their choice. We also suggested the possibility of choosing fractions of days.

## Appendix 5: Practice Sheets Used in Conventional and Chained TTO Interviews

Conventional TTO interview practice sheet example

Chained TTO interview practice sheet example

## Footnotes

1 An example of the set up on the decision board is available in Appendix 6.

2 An A3-sized board on which the EQ-5D profiles were displayed in their participant-ranked order with their corresponding number of days willing to spend in Life B (for the chained version) or days of perfect health given up (for the conventional version). Examples of the Practice sheets are included in Appendix 5.
