# Correction to: Conducting a Time Trade-Off Study Alongside a Clinical Trial: A Case Study and Recommendations

**DOI:** 10.1007/s41669-019-0145-0

**Published:** 2019-05-23

**Authors:** Jing Shen, Sarah Hill, David Mott, Matthew Breckons, Luke Vale, Rob Pickard

**Affiliations:** 1 Health Economics Group, Institute of Health and Society, Newcastle University, Newcastle, UK; 2 Office of Health Economics, London, UK; 3 Institute of Cellular Medicine, Newcastle University, Newcastle, UK

## Correction to: PharmacoEconomics Open (2019) 3:5–20 10.1007/s41669-018-0084-1

The Open Access license, which previously read:

**Open Access** This article is distributed under the terms of the Creative Commons Attribution-NonCommercial 4.0 International License (http://creativecommons.org/licenses/by-nc/4.0/), which permits any noncommercial use, distribution, and reproduction in any medium, provided you give appropriate credit to the original author(s) and the source, provide a link to the Creative Commons license, and indicate if changes were made.

Should read:

**Open Access** This article is distributed under the terms of the Creative Commons Attribution 4.0 International License (http://creativecommons.org/licenses/by/4.0/), which permits unrestricted use, distribution, and reproduction in any medium, provided you give appropriate credit to the original author(s) and the source, provide a link to the Creative Commons license, and indicate if changes were made.

The original article has been corrected.

